# Prognostic value of different sarcopenia assessment methods for mortality in patients with liver cirrhosis: a systematic review and meta-analysis

**DOI:** 10.3389/fnut.2026.1876147

**Published:** 2026-08-13

**Authors:** Wenrui Xie, Wanxin Shi, Runbing Xu, Zimeng Shang, Guangyuan Huo, Zhe Zhang, Zhiyun Yang

**Affiliations:** 1 Center of Integrative Medicine, Beijing Ditan Hospital, Capital Medical University, Beijing, China; 2 Dongzhimen Hospital, Beijing University of Chinese Medicine, Beijing, China; 3 Laboratory for Clinical Medicine, Capital Medical University, Beijing, China

**Keywords:** liver cirrhosis, meta-analysis, mortality, muscle mass, sarcopenia

## Abstract

**Background and Aims:**

Sarcopenia is common among patients with cirrhosis and is closely associated with poor prognosis. However, the value of different methods for assessing sarcopenia in predicting mortality risk in patients with cirrhosis remains controversial. This study aims to systematically evaluate the predictive ability of different methods for measuring sarcopenia regarding mortality risk in patients with cirrhosis.

**Methods:**

A systematic search was conducted in PubMed, Web of Science, and the Embase to identify studies evaluating the association between sarcopenia and the risk of mortality in patients with cirrhosis. The primary outcome measures were the hazard ratio (HR) and its 95% confidence interval. A meta-analysis was performed using a random-effects model, and subgroup analyses were conducted based on different assessment methods, including computed tomography (CT) imaging, handgrip strength (HGS), ultrasound (US), and dual-energy X-ray absorptiometry (DXA).

**Results:**

A total of 15 studies were included. The overall meta-analysis results showed that sarcopenia significantly increased the risk of mortality in patients with cirrhosis. In the CT assessment group, the overall pooled HR was 2.06 (95% CI: 1.65–2.57). Subgroup analysis showed that the HR in the Skeletal Muscle Index (SMI) group was 1.88 (95% CI: 1.50–2.36), the Psoas Muscle Index/Psoas Muscle Thickness/Transverse Psoas Muscle Thickness (PMI/PMT/TPMT) group had an HR of 3.19 (95% CI: 1.62–6.28). The Handgrip Strength (HGS) group had an HR of 2.21 (95% CI: 1.61–3.05), suggesting a significant association between reduced handgrip strength and mortality risk. The HR for the US group was 2.11 (95% CI: 0.75–5.96), which did not reach statistical significance. The HR for the DXA group was 2.38 (95% CI: 1.47–3.84). After harmonizing effect estimates so that lower muscle mass represented the adverse exposure, indicating an increased mortality risk associated with reduced DXA-derived muscle mass. The strength and consistency of evidence varied across different assessment methods.

**Conclusion:**

Sarcopenia is associated with increased mortality risk in patients with cirrhosis. Among currently available assessment approaches, CT-derived measurements, particularly SMI, have the most consistent evidence base for mortality prediction. However, the limited number of studies evaluating HGS, ultrasound, and DXA prevents definitive conclusions regarding the comparative prognostic performance of different methods. Further large-scale prospective studies are required to validate the prognostic value of alternative assessment approaches. Future studies should establish standardized criteria for assessing sarcopenia to improve the accuracy of prognostic evaluations in patients with cirrhosis.

**Systematic review registration:**

https://www.crd.york.ac.uk/prospero, identifier CRD420261334377.

## 1 Introduction

Cirrhosis represents the terminal stage of various chronic liver diseases. Its core pathological features include diffuse liver fibrosis, pseudolobular formation, and intrahepatic vascular proliferation. It can be caused by a variety of factors, including viral hepatitis, alcoholic liver disease, fatty liver disease, and autoimmune liver disease. As a major global public health issue, cirrhosis is associated with high incidence and mortality rates. According to statistics, the prevalence of cirrhosis in the general population worldwide is 1.3% (approximately 112 million cases), while in China, the prevalence is 1.2%, with approximately 10 million patients (1). Once cirrhosis progresses to the decompensated stage, patients are at increased risk of severe complications, including hepatic encephalopathy, variceal bleeding, and hepatorenal syndrome, leading to substantially increased mortality. Currently, the most commonly used prognostic assessment systems for cirrhosis in clinical practice include the Model for End-Stage Liver Disease (MELD) score and the Child-Pugh classification (2). These traditional scoring systems are primarily based on liver function-related indicators. Although they can reflect the severity of a patient’s condition and prognosis to some extent, they have certain limitations and cannot comprehensively cover all key factors influencing patient prognosis. In recent years, with the continuous advancement of clinical research, the role of abnormal body composition (particularly skeletal muscle abnormalities) in the prognostic assessment of patients with cirrhosis has gradually garnered widespread attention. Among these, sarcopenia, as the most common type of abnormal body composition, has become a research hotspot in the field of cirrhosis (3).

Sarcopenia is a syndrome characterized by progressive loss of skeletal muscle mass, decreased muscle strength, and impaired physical function. Although its definition remains controversial, in patients with cirrhosis it typically involves a decline in muscle mass and/or muscle function. The prevalence of this condition is significantly higher in patients with cirrhosis than in the general population, with reported prevalence rates ranging from 30 to 70% across different studies, depending on diagnostic tools and the severity of liver disease. A meta-analysis involving 12,827 patients with cirrhosis showed an overall prevalence of sarcopenia of 44% (4). Importantly, cirrhosis-related sarcopenia is not merely a consequence of malnutrition but rather results from a complex interplay between metabolic disturbances and inflammatory responses associated with chronic liver disease.

The development and progression of sarcopenia in patients with cirrhosis involve multiple pathophysiological mechanisms. Hyperammonemia resulting from impaired hepatic detoxification promotes skeletal muscle catabolism and disrupts mitochondrial function (5). Chronic systemic inflammation further accelerates muscle protein degradation and suppresses protein synthesis through elevated levels of proinflammatory cytokines. In addition, reduced levels of branched-chain amino acids (BCAAs) and disturbances in amino acid metabolism limit the availability of substrates required for muscle protein synthesis. More recently, the concept of the gut–liver–muscle axis has provided further insight into the potential roles of gut microbiota alterations, impaired intestinal barrier function, and dysregulated metabolic signaling in skeletal muscle wasting and functional decline. Collectively, these mechanisms provide a biological rationale for considering sarcopenia an important prognostic factor in cirrhosis (6).

Currently, there are various clinical methods for assessing sarcopenia, each with its own advantages and limitations. These methods can be broadly categorized into three main types: First, CT scans, which serve as a common reference standard for sarcopenia assessment, primarily use metrics such as the skeletal muscle index (SMI), psoas major index (PMI), trunk muscle area (TPMT), to quantitatively assess skeletal muscle mass. It is characterized by high accuracy and strong reproducibility. Both the European Association for the Study of the Liver (EASL) and the American Association for the Study of Liver Diseases (AASLD) recommend it as a key tool for assessing sarcopenia in patients with cirrhosis, and it has been demonstrated that sarcopenia identified by CT is closely associated with patient mortality (7); Second is handgrip strength (HGS) measurement, which primarily reflects muscle function. It is simple to perform, non-invasive, and low-cost, allowing for rapid screening of sarcopenia. Recent studies indicate that handgrip strength may even be a stronger predictor of clinical outcomes than muscle mass (8, 9); third is ultrasound examination, which assesses muscle mass and function by measuring indicators such as muscle thickness and echogenicity. It offers the advantages of being non-invasive, convenient, and suitable for bedside use, making it appropriate for patients who cannot undergo CT scans (3).

Although sarcopenia has been increasingly recognized as an important prognostic factor in cirrhosis, considerable heterogeneity remains regarding diagnostic criteria, cutoff values, and assessment approaches. Previous studies have mainly evaluated individual assessment methods separately, and whether different modalities provide comparable prognostic information remains unclear. In particular, the prognostic value of ultrasound- and DXA-based assessments in cirrhotic populations requires further clarification. Therefore, this systematic review and meta-analysis aimed to comprehensively evaluate the association between different sarcopenia assessment approaches, including CT, HGS, US, and DXA, and mortality risk in patients with cirrhosis. Rather than simply confirming the prognostic significance of sarcopenia, this study aimed to compare the consistency and strength of evidence across different assessment modalities, providing evidence to support clinical risk stratification and future standardization of sarcopenia assessment in cirrhosis.

## 2 Materials and methods

### 2.1 Search strategy and selection criteria

This meta-analysis was conducted in accordance with the latest PRISMA guidelines (2020) (10, 11), and the protocol has been registered on PROSPERO (CRD420261334377). We systematically searched databases including PubMed, Web of Science, Embase, CNKI, and Wanfang Database from inception through December 31, 2025, to identify relevant full-text studies examining the relationship between various measures of sarcopenia and survival outcomes in patients with cirrhosis.

Search terms were carefully designed based on the research topic and Medical Subject Headings (MeSH), incorporating both core and expanded keywords to enhance the comprehensiveness of the search. Core English keywords included “liver cirrhosis,” “sarcopenia,” and “mortality,” while expanded keywords included “hepatic cirrhosis,” “muscle loss,” “skeletal muscle wasting,” “survival,” “prognosis,” “outcome,” “all-cause mortality,” and “liver-related mortality.” The search strategy combines subject headings with free-text terms, using the Boolean operators “AND” and “OR” to create combinations, and adjusts the specific search queries according to the retrieval rules of each database to ensure the accuracy and comprehensiveness of the search results. For example, the search query for the PubMed database was: (“liver cirrhosis” OR “hepatic cirrhosis”) AND (“sarcopenia” OR “muscle loss” OR “skeletal muscle wasting”) AND (“mortality” OR “survival” OR “prognosis” OR “death risk”). Supplementary Appendix 1 shows the search strategies for all included databases.

### 2.2 Data extraction and quality assessment

We used a standardized data extraction form to extract data from each included study. The following information was independently extracted by two reviewers: the first author’s name, year of publication, study design, study location, inclusion and exclusion criteria (Supplementary Appendix 2), methods used to measure muscle mass or muscle function, number of participants, and patient demographics and clinical characteristics, including age, sex, cause of cirrhosis, duration of follow-up, adjustment variables, and relevant outcomes. If relevant data were not readily available, the authors were contacted to obtain additional data and/or clarification.

The quality of included studies was also scored by at least 2 authors (XWR and SWX) independently using a scale based on the Newcastle-Ottawa scale (NOS) (12), with disagreements resolved by consensus or discussion with a third author (XRB). The detail of assessment is described in Supplementary Appendix 3. The GRADE approach was used to assess the evidence on prognosis (13) (Supplementary Table 1).

### 2.3 Statistical analysis

The statistical analysis for this study was conducted using R software (14). All statistical tests were two-sided; *P* < 0.05 was considered statistically significant. The primary outcome measures in this study were the hazard ratio (HR) and its 95% confidence interval (95% CI). If a study directly provided the HR and its 95% CI, the multivariately adjusted HR was extracted; if not, it was calculated based on the raw data or derived from the log-transformed HR (logHR) and standard error (SE). Due to differences among included studies in baseline patient characteristics, definitions of sarcopenia, and follow-up durations, a random-effects model (DerSimonian–Laird method) (15) was used for meta-analysis. Heterogeneity among studies was assessed using I^2^ and Cochran’s Q statistic. Significant heterogeneity was defined as a *P*-value < 0.10 for the Q-test or *I*^2^ > 50%. To compare the differences in the predictive ability of various sarcopenia measurement methods for mortality risk, the studies were categorized into four groups: CT, HGS, US, and DXA. Within the CT assessment group, further subgroup analyses were conducted based on specific muscle indices, including SMI, PMI, PMT, and TPMT. Differences between subgroups were compared using the χ^2^ test to determine whether statistically significant differences existed among the different measurement methods. To ensure consistency in the interpretation of effect estimates across different sarcopenia assessment modalities, all hazard ratios were harmonized to represent the mortality risk associated with reduced muscle mass or impaired muscle function compared with preserved muscle status. For continuous variables where higher values indicated better muscle status (e.g., DXA-derived lean mass indices or ultrasound-measured muscle thickness), hazard ratios were transformed using the reciprocal method (HR′ = 1/HR), with corresponding confidence intervals inverted accordingly. A leave-one-out analysis was performed to assess the impact of individual studies on the overall results and to verify the stability of the findings. Additionally, studies that reported only multivariately adjusted HRs were reanalyzed to minimize the influence of confounding factors. Publication bias was assessed using funnel plots, Egger’s regression test, and Begg’s rank correlation test when at least 10 independent effect estimates were available. For subgroup analyses with fewer than 10 studies, these tests were not performed because of insufficient statistical power. When funnel plot asymmetry was observed, a trim-and-fill analysis was conducted to evaluate the potential influence of missing studies. The Newcastle–Ottawa Scale (NOS) (12) was used to assess the quality of the included studies, with scoring based on three dimensions: study selection, comparability, and outcome assessment. The total score ranged from 0 to 9 points, with a score of ≥7 indicating high study quality.

## 3 Results

### 3.1 Study retrieval characteristics of included studies

**FIGURE 1:**
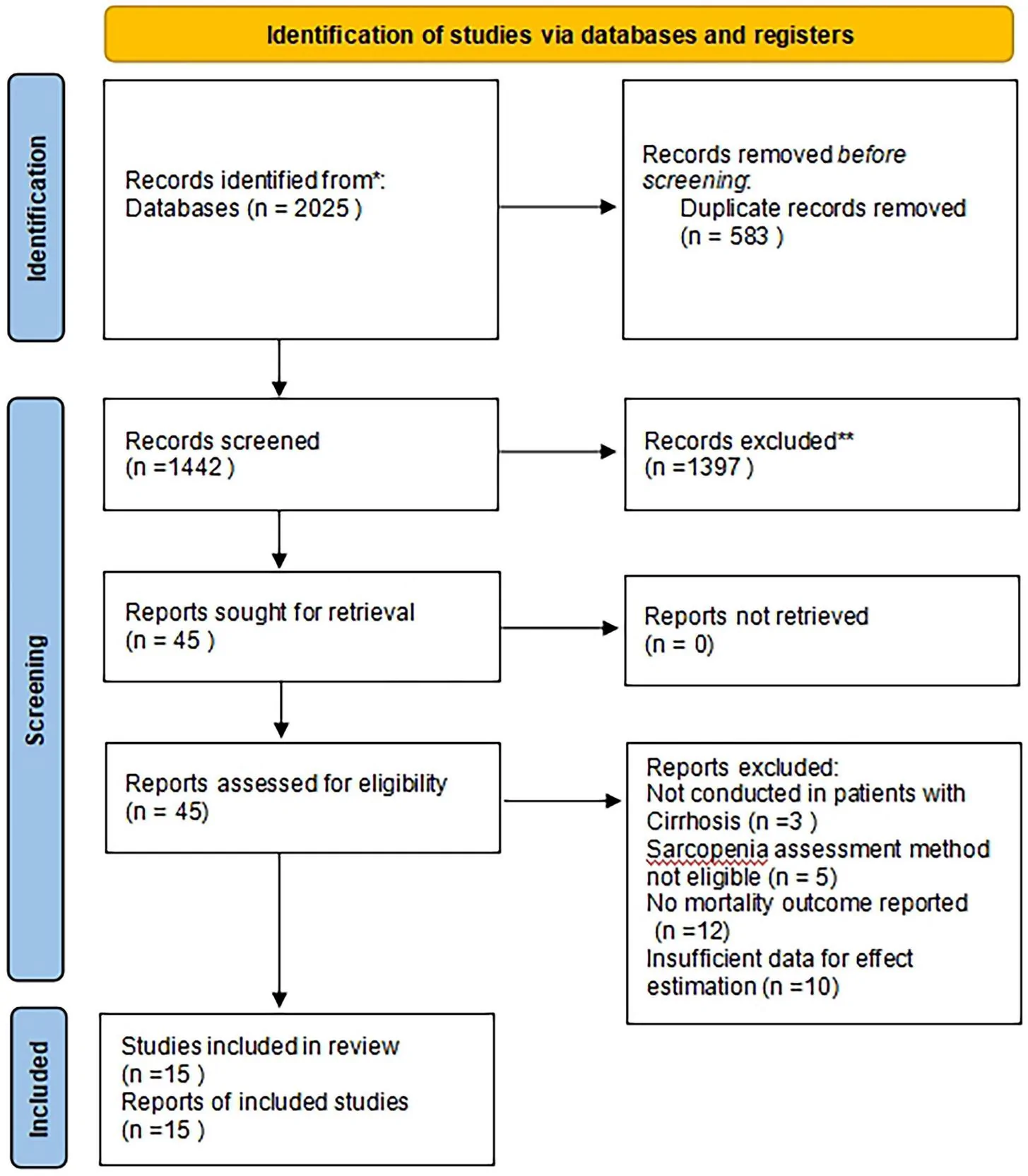
PRISMA flow diagram of search strategy.

Of the 2,025 records identified in the initial search, 583 duplicates and 1,407 ineligible records were excluded. Of the remaining 45 articles that underwent full-text screening, we included 15 cohort studies comprising data from 5,208 patients (Figure 1). Table 1 summarizes the characteristics of all studies included in this meta-analysis. Of the 15 studies (16, 17), 9 were from Asia (16, 18–21) and 6 were from non-Asian regions (17, 22–24) Among the 15 studies, 8 were retrospective cohort studies (16, 20–22, 24, 25), and 7 were prospective cohort studies (17, 18, 23, 26, 27). The sample sizes of the included studies ranged from 1,624 to 61, and the mean age of patients ranged from 50 to 80.4 years. All studies were rated as high quality, with a NOS score of ≥7 (Supplementary Table 2). Detailed characteristics of sarcopenia assessment methods and extracted effect estimates are presented in Supplementary Tables 3, 4.

**TABLE 1:** Summary characteristics of included studies.

No.	References	Country	Methods for sarcopenia assessment	Sample size	Age	Male	Liver disease stage	Follow-up	Outcome	Deaths
1	Gu et al. (16)	South Korea	CT(L3-PMTH)	653	53.6 ± 10.2	76.40%	Cirrhosis	11.9 ± 5.6 months	All-cause mortality	21
2	Anand et al. (26)	India	CT(L3-SMI)	219	42.6 ± 10.0	76.70%	Cirrhosis	Median 12 months	All-cause mortality	30
3	Thyloor Kenchappa et al. (35)	India	CT(L3-PMI)	156	50	72%	Cirrhosis	6 months	All-cause mortality	21
4	Sun et al. (36)	China	CT(L3-SMI)	478	64 ± 7	46.70%	Cirrhosis	24 months	All-cause mortality	118
5	Rodge et al. (18)	India	CT(L3-PMI)	75	52.2 ± 7.9	54.70%	Chronic liver disease	12 months	All-cause mortality	9
6	Moctezuma-Velazquez et al. (22)	Canada	CT(L3-SMI)	315	54 ± 8	66%	Cirrhosis	Median 10.9 months	All-cause mortality	109
7	Cho et al., 2021 (19)	South Korea	CT(L3-SMI)	166	53.8 ± 10.4	70.50%	Cirrhosis	Median 38.6 months	All-cause mortality	50
8	Hanai et al. (37)	Japan	HGS	563	71 ± 11	67%	Cirrhosis	26 months	All-cause mortality	207
9	Nishikawa et al. (20)	Japan	HGS	1624	57.5 ± 36.5	60.10%	Chronic liver disease	Median 712 days	All cause mortality	354
10	Ciocîrlan et al. (23)	Romania	US	61	58.0 ± 10.8	67%	Cirrhosis	11.9 ± 5.6 months	All-cause mortality	18
11	Kikuchi et al. (21)	Japan	CT(L3-SMI)	300	69.1 ± 11.3	58%	Cirrhosis	36 months	All-cause mortality	72
12	Eriksen et al. (24)	Denmark	DXA	315	59.35 ± 7.25	73.30%	Cirrhosis	Median 55 months	All cause mortality	166
13	Belarmino et al. (27)	Brazil	DXA	129	54 ± 9.9	100%	Cirrhosis	36 months	All-cause mortality	55
14	Ayadi et al. (25)	Tunisia	CT(L3-TPMT)	70	62 ± 12	44%	Cirrhosis	Median 21 months	All-cause mortality	29
15	De Monte et al. (17)	Germany	US	84	60	60%	Cirrhosis	6 months	All-cause mortality	13

L3, 3rd lumbar vertebra; HGS, handgrip strength; US, Ultrasound Scanner; DXA, dual-energy x-ray absorptiometry; PMI, psoas muscle index; SMI, skeletal muscle index; TPMT, transverse psoas muscle thickness; PMTH, Psoas Muscle Thickness to Height Ratio.

### 3.2 Quality of study

**FIGURE 2:**
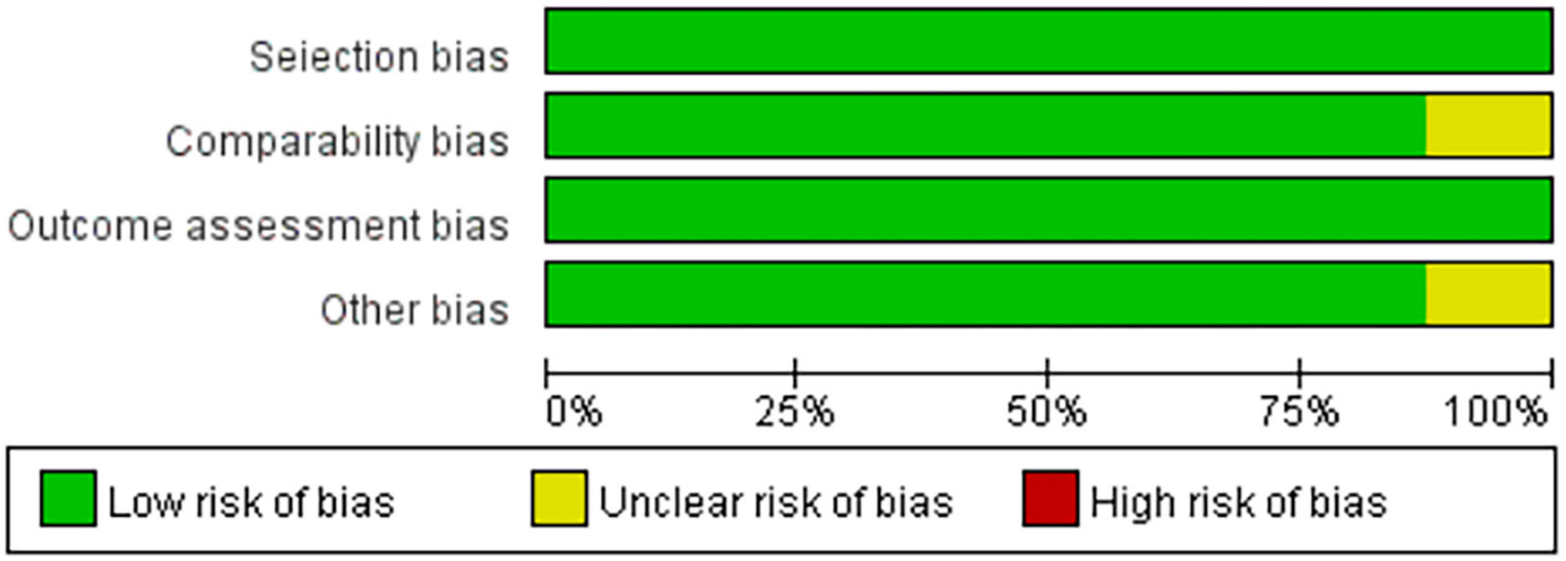
Methodological quality assessment of the risk of bias for each included study.

The methodological quality of the included studies was assessed using the Newcastle–Ottawa Scale (NOS). This scale evaluates study quality based on three criteria—selection, comparability, and outcome assessment—with a total score ranging from 0 to 9. A total of 15 studies were included in this analysis; the NOS scores showed that all studies scored ≥7, indicating a high overall methodological quality. Most studies received high scores in the areas of participant selection and outcome assessment, indicating that the source of participants was well-defined and that follow-up outcome assessments were reliable. Regarding comparability between study groups, some studies adjusted for potential confounding factors such as age, sex, cause of liver disease, or disease severity using multivariate regression models; however, a few studies did not perform adequate adjustments. The risk of bias assessment showed that most studies exhibited a low risk of bias across all evaluation dimensions. Overall, the included studies demonstrated good methodological quality, and the study results are highly credible. Specific NOS scores and risk of bias assessments are presented in Figure 2 and Supplementary Table 2.

### 3.3 Sarcopenia assessed by CT and mortality risk in patients with cirrhosis

**FIGURE 3:**
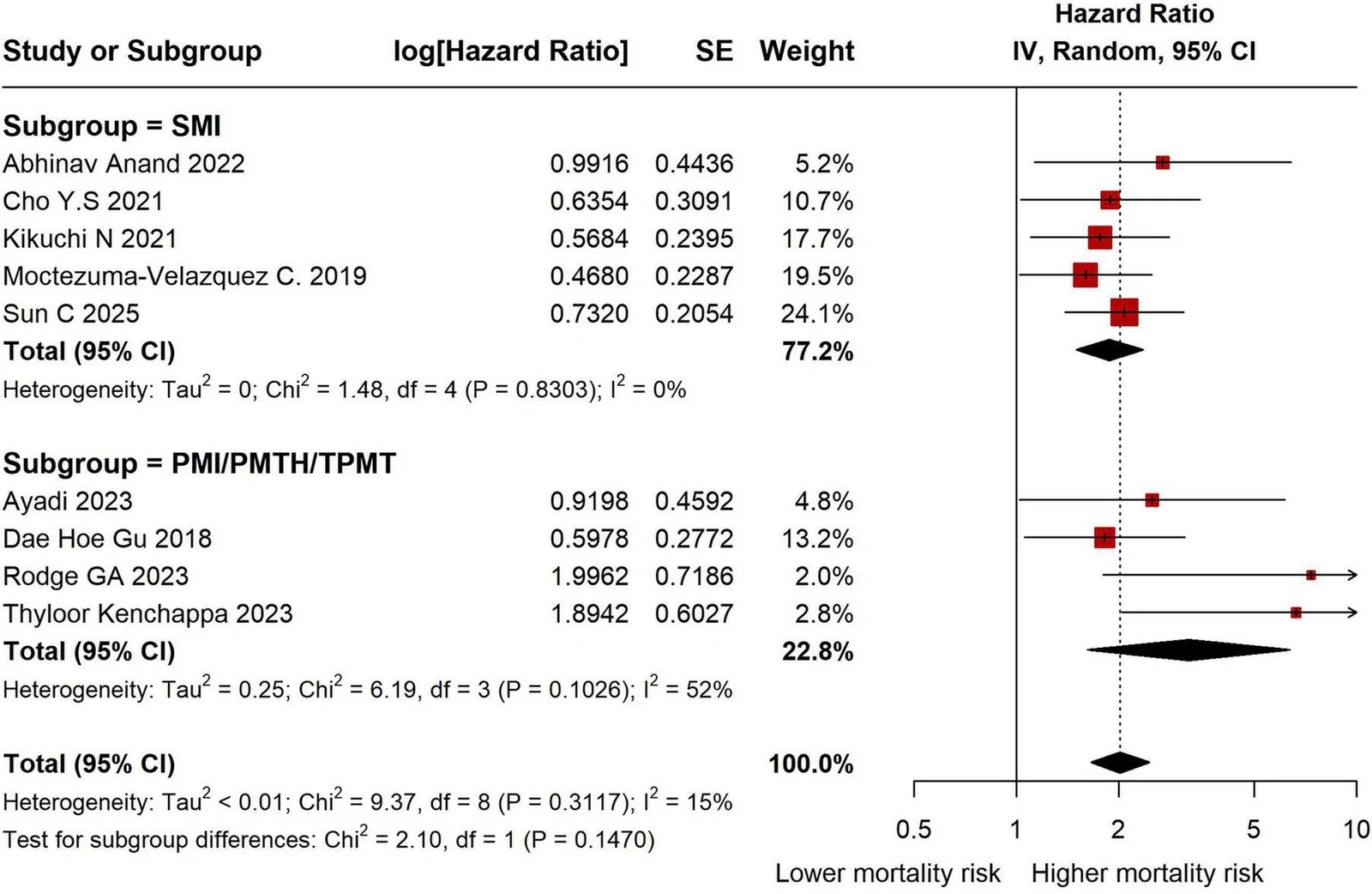
Forest plot of the association between CT-assessed sarcopenia and mortality in patients with liver cirrhosis.

This study included a total of 9 studies that used CT imaging methods to assess sarcopenia (Figure 3). Based on differences in specific measurement indices, the CT assessment group was further divided into a skeletal muscle index (SMI) subgroup and a psoas-related index (PMI/PMT/TPMT) subgroup for stratified analysis. The SMI subgroup included 5 studies. A meta-analysis using a random-effects model showed that sarcopenia was significantly associated with mortality risk in patients with cirrhosis (HR = 1.88, 95% CI: 1.50–2.36, *P* < 0.00001). Heterogeneity among studies was low (*I*^2^ = 0%, *P* = 0.83), suggesting that SMI measured by CT can effectively predict the risk of mortality in patients with cirrhosis. The PMI/PMT/TPMT subgroup included 4 studies. The results of the meta-analysis showed that these psoas-related indices were also significantly associated with the risk of mortality in patients with cirrhosis (HR = 3.19, 95% CI: 1.62–6.28, *P* = 0.0008), with moderate heterogeneity among studies (*I*^2^ = 52%, *P* = 0.10). The overall pooled analysis of CT-based assessments showed that CT-assessed sarcopenia significantly increased the risk of mortality in patients with cirrhosis (HR = 2.06, 95% CI: 1.65–2.57, *P* < 0.00001), with low heterogeneity among studies (*I*^2^ = 15%, *P* = 0.31); Subgroup analysis results indicated (*P* = 0.15) that there was no statistically significant difference in the predictive value for mortality risk in patients with cirrhosis between SMI and the two CT measurement methods (PMI/PMT/TPMT).

### 3.4 HGS and mortality risk in patients with cirrhosis

**FIGURE 4:**
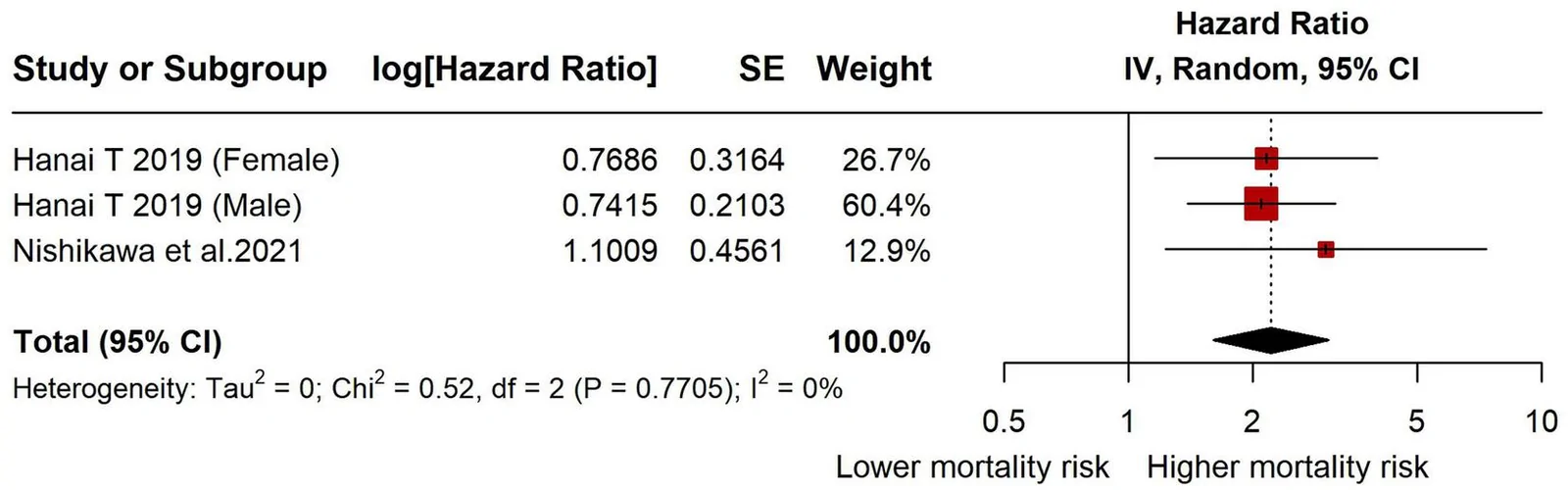
Forest plot of the association between hand grip strength (HGS) and mortality in patients with liver cirrhosis.

This study included a total of 3 studies that used handgrip strength to assess sarcopenia (Figure 4). A meta-analysis using a random-effects model revealed that sarcopenia characterized by reduced handgrip strength was significantly associated with an increased risk of mortality in patients with cirrhosis (HR = 2.21, 95% CI: 1.61–3.05, *P* < 0.00001). Furthermore, heterogeneity among the included studies was low (*I*^2^ = 0%, *P* = 0.77), suggesting that sarcopenia assessed by grip strength may serve as an effective predictor of mortality risk in patients with cirrhosis.

### 3.5 Muscle thickness assessed by US and risk of mortality

**FIGURE 5:**
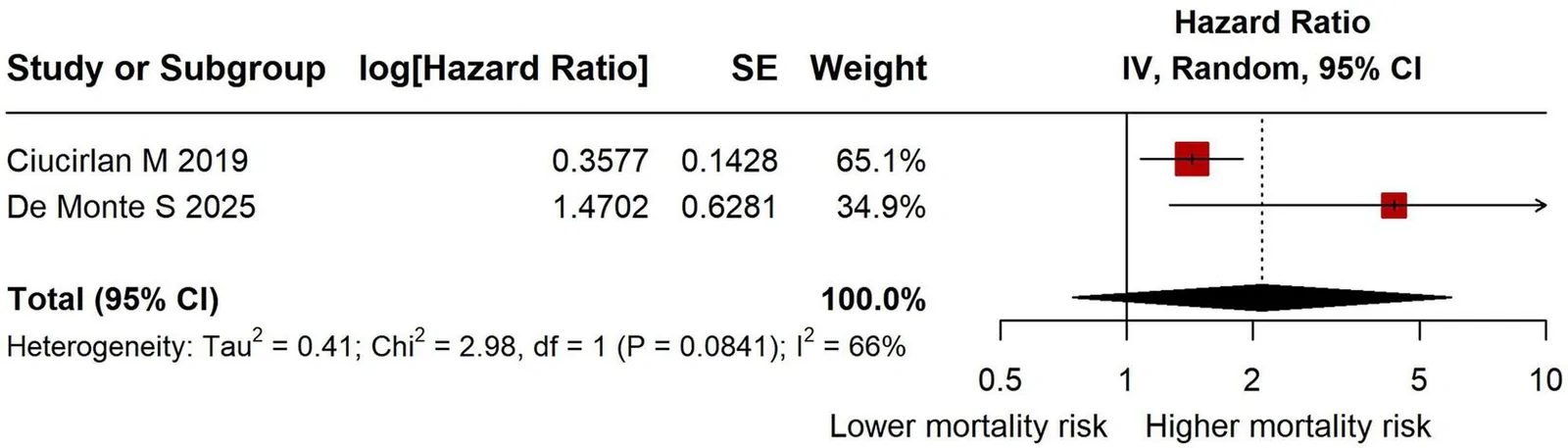
Forest plot of the association between ultrasound-assessed muscle thickness and mortality in patients with liver cirrhosis.

This study included two studies that used ultrasound-measured muscle thickness for the assessment of sarcopenia (Figure 5). After harmonizing the direction of effect estimates, with lower muscle thickness consistently representing impaired muscle status, the random-effects meta-analysis showed that reduced ultrasound-measured muscle thickness was associated with an increased risk of mortality in patients with cirrhosis, although the association did not reach statistical significance (HR = 2.11, 95% CI: 0.75–5.96, *P* = 0.16). Moderate-to-high heterogeneity was observed among the included studies (*I*^2^ = 66%, *P* = 0.08), which may be related to differences in ultrasound measurement sites, assessed muscle groups, and cutoff definitions. The association between ultrasound-measured muscle thickness and mortality was not statistically significant, with substantial heterogeneity among studies. Given the limited number of available studies and variability in ultrasound measurement protocols, these findings should be interpreted cautiously and require further validation.

### 3.6 Sarcopenia assessed by DXA and risk of mortality

**FIGURE 6:**
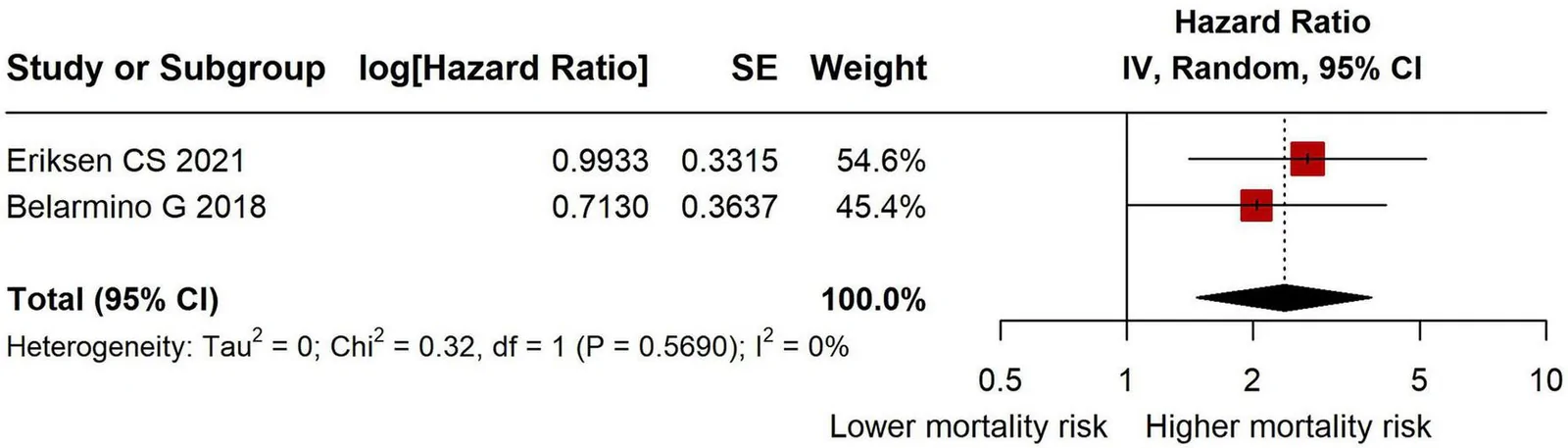
Forest plot of the association between DXA-assessed muscle mass and mortality in patients with liver cirrhosis.

This study included two studies that used DXA-derived skeletal muscle mass indices for the assessment of sarcopenia (Figure 6). After harmonizing the direction of effect estimates, where lower muscle mass was defined as the adverse exposure, the random-effects meta-analysis demonstrated that reduced DXA-derived skeletal muscle mass was significantly associated with increased mortality risk in patients with cirrhosis (HR = 2.38, 95% CI: 1.47–3.84, *P* < 0.001). Heterogeneity among the included studies was low (*I*^2^ = 0%, *P* = 0.57), suggesting consistent findings across the available DXA-based studies. However, because only two studies were available, these results should be interpreted as supportive evidence rather than definitive proof of the prognostic superiority of DXA-based assessment.

### 3.7 Publication bias and sensitivity analysis

Publication bias was assessed for the overall meta-analysis because the number of included effect estimates was sufficient for statistical evaluation. The funnel plot showed some asymmetry (Supplementary Figure 1). Egger’s regression test and Begg’s rank correlation test both indicated potential publication bias (Egger’s test: *t* = 6.65, *P* < 0.0001; Begg’s test: *z* = 3.33, *P* = 0.0009). Therefore, a trim-and-fill analysis was further performed to explore the potential influence of missing studies. The adjusted pooled estimate remained consistent with the original analysis, suggesting that the overall association between sarcopenia and mortality risk was not substantially altered by potential publication bias (Supplementary Figure 1). Leave-one-out sensitivity analysis demonstrated that exclusion of any single study did not materially change the pooled hazard ratio, with the pooled estimates remaining within a relatively narrow range (HR range: 1.89–2.11), indicating the robustness of the primary findings (Supplementary Figure 2).

## 4 Discussion

### 4.1 Key findings

This study conducted a systematic review of the predictive value of various methods for assessing sarcopenia on the risk of mortality in patients with cirrhosis. The results of the meta-analysis indicate that sarcopenia is significantly associated with the risk of mortality in patients with cirrhosis. Overall analysis results indicate that sarcopenia significantly increases the risk of mortality in patients with cirrhosis. In subgroup analyses of different assessment methods, CT imaging, handgrip strength (HGS) and DXA-derived muscle mass measurements all demonstrated significant associations with mortality risk in the available studies, whereas ultrasound measurement of muscle thickness did not reach statistical significance. Specifically, the overall hazard ratio (HR) for sarcopenia assessed by CT was 2.06 (95% CI: 1.65–2.57), suggesting that a decrease in muscle mass measured by CT is associated with a significantly increased risk of mortality. In the CT subgroup analysis, both the SMI and PMI/PMT/TPMT indices demonstrated strong predictive ability. Furthermore, grip strength, as a functional indicator, was also significantly associated with mortality risk. In contrast, results from ultrasound-measured muscle thickness did not reach statistical significance and exhibited high heterogeneity. Overall, the findings suggest that different sarcopenia assessment approaches demonstrate varying degrees of association with mortality risk in patients with cirrhosis. CT-derived indices, particularly SMI, showed relatively consistent associations across multiple cohorts. However, because the number of studies included in several modality-specific subgroups was limited, especially for HGS, ultrasound, and DXA, direct comparisons of predictive performance between methods should be interpreted cautiously.

### 4.2 Evidence regarding different sarcopenia assessment approaches and mortality risk

CT is currently the most commonly used method for assessing sarcopenia in research. CT imaging can accurately measure skeletal muscle area at the L3 level and calculate the skeletal muscle index (SMI) through standardized calculations; it is considered the “gold standard” for assessing muscle mass. The results of this study indicate that sarcopenia assessed by CT is significantly associated with mortality risk, and the low heterogeneity across studies suggests that this method possesses good stability and reproducibility. In the CT subgroup analysis, the heterogeneity for the SMI subgroup was 0%, indicating a high degree of consistency in results across different studies. This may be related to the high degree of standardization in the SMI measurement method. The PMI/PMT/TPMT subgroup included a total of 4 studies. The pooled analysis results showed that these psoas-related indices were also significantly associated with the risk of mortality in patients with cirrhosis (HR = 3.19, 95% CI: 1.62–6.28, *P* = 0.0008), with moderate heterogeneity among studies (*I*^2^ = 52%, *P* = 0.10). This heterogeneity may be related to differences in the measurement methods and diagnostic criteria for psoas major indices across studies. Compared with SMI, PMI, PMT, and TPMT are all indices based on local measurements of the psoas major muscle; differences exist among studies regarding the measurement plane, normalization methods (e.g., height- or body surface area-adjusted), and cutoff value settings. Furthermore, differences in the etiology of cirrhosis, disease severity, and follow-up duration among the study populations may also exist. These factors could influence mortality risk, thereby contributing to a certain degree of heterogeneity among the study results. Nevertheless, the direction of effect sizes was generally consistent across studies, and the results of the meta-analysis remained statistically significant, suggesting that psoas-related indices still hold clinical value in assessing sarcopenia and predicting mortality risk in patients with cirrhosis. The incidence of sarcopenia is high among patients with cirrhosis, and its pathogenesis may be associated with factors such as chronic inflammation, abnormal protein metabolism, inadequate nutritional intake, and altered hormone levels. A reduction in muscle mass not only affects metabolic function but may also lead to impaired immune function, thereby increasing the risk of infections, hepatic encephalopathy, and other complications, ultimately resulting in increased mortality.

HGS is an important indicator for assessing muscle function and is one of the diagnostic criteria recommended by the European Working Group on Sarcopenia (EWGSOP). The results of this study indicate that a decline in hand grip strength is significantly associated with an increased risk of mortality in patients with cirrhosis. Compared to imaging methods, hand grip strength measurement offers advantages such as simplicity, low cost, and high reproducibility, making it highly valuable for clinical practice. Hand grip strength not only reflects muscle strength but also, to some extent, reflects the patient’s overall physical functional status. Previous studies have shown that a decline in muscle strength often precedes a reduction in muscle mass; therefore, hand grip strength may serve as a sensitive indicator for predicting poor patient outcomes.

Ultrasound measurement of muscle thickness is a non-invasive and convenient assessment method; After harmonizing the direction of effect estimates, reduced ultrasound-measured muscle thickness showed a trend toward increased mortality risk in patients with cirrhosis, although the pooled association did not reach statistical significance (HR = 2.11, 95% CI: 0.75–5.96). Moderate-to-high heterogeneity was observed among the included studies (*I*^2^ = 66%), suggesting substantial methodological and clinical variability. Several factors may contribute to this heterogeneity. First, the anatomical sites assessed by ultrasound were not consistent across studies. Specifically, one study evaluated rectus abdominis muscle thickness, whereas another assessed rectus femoris muscle thickness. These different muscle groups may reflect distinct aspects of skeletal muscle depletion and may not be directly comparable. Second, ultrasound measurement protocols, including scanning planes, probe positioning, and operator-dependent procedures, may vary across studies, potentially affecting measurement reproducibility (28). Third, different cutoff values were applied to define reduced muscle mass, and standardized ultrasound-based diagnostic thresholds for sarcopenia in patients with cirrhosis have not yet been established. Importantly, after harmonization of effect directions, the included studies showed generally consistent associations between reduced muscle thickness and increased mortality risk, suggesting that the observed heterogeneity was more likely attributable to methodological differences rather than conflicting biological effects. In addition, ultrasound primarily captures localized muscle characteristics rather than overall skeletal muscle mass, which may further contribute to variability among studies. Given the limited number of available studies and methodological heterogeneity, the prognostic value of ultrasound-based sarcopenia assessment remains uncertain and requires further validation in large-scale prospective studies using standardized protocols.

DXA is a widely used technique for evaluating body composition and provides quantitative measurements of skeletal muscle mass. After harmonizing the direction of effect estimates, reduced DXA-derived muscle mass was significantly associated with increased mortality risk in patients with cirrhosis (HR = 2.38, 95% CI: 1.47–3.84). The low heterogeneity observed among the available studies (*I*^2^ = 0%) suggests relatively consistent findings across DXA-based cohorts. However, only two studies were available for this subgroup analysis, and the prognostic value of DXA-based assessments should therefore be interpreted cautiously. Several factors may affect the applicability of DXA-based assessment in patients with cirrhosis. First, DXA evaluates body composition through measurements of lean mass rather than directly assessing muscle strength or physical function, which may not fully capture the multidimensional characteristics of sarcopenia. Second, fluid-related complications, including ascites and peripheral edema, are common in patients with advanced cirrhosis and may influence body composition measurements, potentially leading to inaccurate estimation of skeletal muscle mass (29). Third, different DXA-derived parameters were used across studies, including variations in the definition of low muscle mass, which may limit direct comparability. Therefore, although the current findings suggest that reduced DXA-derived muscle mass is associated with increased mortality risk, the available evidence remains insufficient to determine whether DXA provides superior prognostic information compared with other assessment approaches. Further studies are needed to determine the role of DXA-based muscle assessment in clinical risk stratification.

### 4.3 Strengths and limitation

The findings of this study have important clinical implications. Sarcopenia represents an important prognostic marker in patients with cirrhosis and may provide additional information beyond traditional liver function-based prognostic models. In particular, CT-defined sarcopenia was associated with approximately a two-fold increased risk of mortality, suggesting that patients with significant skeletal muscle depletion may represent a high-risk subgroup requiring closer clinical attention. From a clinical management perspective, identification of sarcopenia may help improve risk stratification and guide individualized patient management. Patients with cirrhosis and confirmed muscle depletion may benefit from more frequent nutritional assessment, early implementation of nutritional interventions, and multidisciplinary management involving hepatologists, dietitians, and rehabilitation specialists. In addition, functional assessment using tools such as handgrip strength may complement imaging-based evaluation by providing information regarding muscle performance and physical function. However, the presence of sarcopenia should not be considered an independent indication for specific therapeutic decisions. Instead, sarcopenia assessment should be integrated with established prognostic systems, such as MELD and Child–Pugh scores, to provide a more comprehensive evaluation of disease severity and mortality risk. Future prospective intervention studies are required to determine whether sarcopenia-guided nutritional and rehabilitation strategies can improve clinical outcomes.

The certainty of evidence was evaluated using the GRADE approach (Supplementary Table 2), and the overall quality ranged from low to high depending on the sarcopenia assessment method. CT-derived SMI demonstrated high-certainty evidence, supported by consistent findings and minimal heterogeneity across studies. In contrast, psoas muscle-related indices (PMI/PMT/TPMT) and HGS were supported by moderate-certainty evidence, primarily due to limited sample size and moderate heterogeneity. Ultrasound-based measurements showed low-certainty evidence, because of the limited number of studies and methodological heterogeneity. Similarly, DXA-based assessments were graded as low-certainty evidence due to the small number of included studies and potential publication bias. These findings suggest that CT-based measurements, particularly SMI, are supported by the most consistent evidence for mortality risk assessment, whereas other methods require further validation in larger and more standardized studies.

However, this study has certain limitations. First, diagnostic criteria for sarcopenia varied across studies. For example, the cutoff values for SMI were inconsistent across studies, which may affect the comparability of the results (30). Second, Another important limitation is that the limited number of included studies prevented us from conducting detailed subgroup analyses or meta-regression analyses to explore potential sources of clinical heterogeneity. Several clinically relevant factors, including age, sex, follow-up duration, severity of liver disease (e.g., MELD score and Child–Pugh class), cirrhosis etiology, presence of ascites, hepatocellular carcinoma, and liver transplantation status, may influence both the prevalence of sarcopenia and its association with mortality (9). In particular, variations in follow-up duration across studies may reflect different clinical scenarios, as short-term mortality may be driven predominantly by acute decompensation events, whereas long-term mortality may better capture cumulative effects of nutritional decline, disease progression, and chronic complications. Therefore, the pooled estimates should be interpreted cautiously, and future studies with larger sample sizes and standardized follow-up periods are needed to clarify these potential modifiers. Furthermore, the ultrasound subgroup did not reach statistical significance and exhibited high heterogeneity; the current evidence is insufficient to support ultrasound muscle thickness as a reliable prognostic predictor. It should be noted that the included studies in this review originated from different study populations, making it impossible to test for differences between methods. Additionally, some subgroups had a small number of included studies, particularly the DXA and US groups, which may affect the stability of the pooled results. the limited number of studies in several subgroups, particularly HGS, ultrasound, and DXA, may have reduced statistical power and increased uncertainty. Therefore, conclusions regarding the comparative prognostic value of different sarcopenia assessment methods should be considered hypothesis-generating rather than definitive. In addition, several alternative approaches for sarcopenia assessment, including bioelectrical impedance analysis (BIA), magnetic resonance imaging (MRI), calf circumference, SARC-F questionnaire, and gait speed, were not included in the present meta-analysis (31). These methods were excluded because available studies assessing mortality outcomes in patients with cirrhosis were limited, or because the reported measurements were not directly comparable with the included modalities. Future studies are needed to determine whether these alternative approaches provide additional prognostic information beyond currently established assessment methods. Finally, although additional searches of Chinese databases, including China National Knowledge Infrastructure (CNKI) and Wanfang Database, were conducted, no Chinese-language studies meeting the inclusion criteria and reporting mortality outcomes were identified. Therefore, all included studies were derived from international databases. Although this additional search reduced the possibility of language-related omission, potential database selection bias cannot be completely excluded.

### 4.4 Future research directions

Future research should focus on conducting large-scale, multicenter prospective studies to validate the role of different methods for assessing sarcopenia in predicting the prognosis of patients with cirrhosis. At the same time, it is necessary to establish uniform diagnostic criteria for sarcopenia to improve comparability across different studies. Furthermore, future research could explore the value of combined assessments of imaging and functional indicators in predicting the prognosis of patients with cirrhosis. For example, combining muscle mass measured by CT or DXA with functional indicators such as grip strength may help provide a more comprehensive evaluation of patients’ nutritional status and risk of disease progression. In addition to imaging-based and functional assessments, emerging molecular approaches may provide new opportunities for sarcopenia diagnosis and risk stratification. Recent studies have explored the potential of metabolomic and proteomic signatures as non-invasive biomarkers for identifying individuals with reduced skeletal muscle mass. For example, a recent study published in *Nature Aging* developed a metabolomics-based approach for diagnosing sarcopenia and predicting skeletal muscle index (SMI), highlighting the potential application of molecular profiling in muscle health assessment (32, 33). These approaches may complement conventional assessment methods by capturing biological processes associated with muscle loss, including inflammation, mitochondrial dysfunction, amino acid metabolism, and energy imbalance. However, their clinical utility remains to be validated in diverse cirrhotic populations, and future prospective studies are required to determine whether molecular biomarkers can improve mortality prediction beyond established imaging and functional assessments. Of course, With the advancement of artificial intelligence and image analysis technologies, automated muscle measurement methods may offer new research directions for sarcopenia assessment. For instance, measuring muscle mass in CT images using automated image analysis technology can improve assessment efficiency and reduce human error (34). Furthermore, incorporating sarcopenia into existing liver cirrhosis prognostic scoring systems (such as the MELD score) may help further improve the accuracy of risk stratification for patients with liver cirrhosis.

## Data Availability

The original contributions presented in this study are included in this article/Supplementary material, further inquiries can be directed to the corresponding author.
