# Supplementary material for: Prognostic value of different sarcopenia assessment methods for mortality in patients with liver cirrhosis: a systematic review and meta-analysis
Source: Front Nutr. 2026 Aug 13;13:1876147. doi: 10.3389/fnut.2026.1876147 (PMC13518161; doi:10.3389/fnut.2026.1876147)
Supplement: Supplementary file 1 [file Table_1.docx]

Supplementary Material

# 1 Supplementary Data

**Appendix1.**Detailed search strategy and process

**Pubmed：**

1.(cirrhosis[Title/Abstract]) OR (liver cirrhosis[Title/Abstract]) OR (liver disease[Title/Abstract])

2.(skeletal muscle[Title/Abstract]) OR (muscle index[Title/Abstract]) OR (psoas[Title/Abstract]) OR (muscle loss[Title/Abstract]) OR (skeletal muscle[Title/Abstract]) OR (grip strength[Title/Abstract]) OR (handgrip strength[Title/Abstract]) OR (muscle index[Title/Abstract]) OR (muscular weakness[Title/Abstract])

3.(death[Title/Abstract]) OR (outcome[Title/Abstract]) OR (mortality[Title/Abstract]) OR (survival[Title/Abstract])

1 AND 2 AND 3

**Web of science:**

(“Cirrhosis” OR “liver disease” OR “liver cirrhosis”) AND (“sarcopenia” OR “muscle mass” OR “skeletal muscle” OR “psoas” OR “muscle index” OR “muscular weakness” OR “handgrip strength”OR “grip strength”OR “Hand Strength” ) AND (“mortality”OR “death” OR “outcome”)

**Embase:**

1.('muscle mass'/exp) OR ('skeletal muscle'/exp) OR ('psoas muscle'/exp) OR ('muscle strength'/exp) OR ('muscle weakness'/exp) OR ('grip strength'/exp) OR ('hand strength'/exp) OR ('sarcopeni*':ti,ab) OR ('muscle loss':ti,ab) OR ('psoas muscle':ti,ab) OR ('muscle mass':ti,ab) OR ('skeletal muscle':ti,ab) OR ('handgrip strength':ti,ab) OR ('grip strength':ti,ab) OR ('hand strength':ti,ab)

2.('liver cirrhosis'/exp) OR ('cirrhosis':ti,ab) OR ('cirrhotic':ti,ab) OR ('liver disease*':ti,ab) OR ('liver fibrosis':ti,ab)

3.('death'/exp) OR ('mortality'/exp) OR ('survival'/exp) OR ('prognosis'/exp) OR ('outcome*':ti,ab) OR ('death*':ti,ab) OR ('mortality':ti,ab) OR ('surviv*':ti,ab) OR ('prognos*':ti,ab)

1 AND 2 AND 3

**Appendix2.Inclusion and exclusion criteria for study selection**

Studies were included if they met the following criteria:

(1)**Population:** Adult patients (≥18 years) diagnosed with liver cirrhosis regardless of etiology.

(2)**Exposure:** Sarcopenia assessed using recognized measurement methods, including computed tomography (CT)-derived muscle indices (e.g., skeletal muscle index [SMI] or psoas muscle-related indices such as PMI/PMT/TPMT), hand grip strength (HGS), ultrasound (US) measurements of muscle thickness, or dual-energy X-ray absorptiometry (DXA).

(3)**Outcome:** Studies reporting mortality outcomes (all-cause mortality) in patients with liver cirrhosis.

(4)**Effect size:** Studies providing hazard ratios (HRs) with 95% confidence intervals (CIs), or sufficient data to calculate HRs.

(5)**Study design:** Observational studies, including prospective cohort studies, retrospective cohort studies, or case-control studies.

Studies were excluded if they met any of the following criteria:

(1)Studies that did not include patients with confirmed liver cirrhosis or did not report data specifically for cirrhotic patients.

(2)Studies that did not assess sarcopenia or skeletal muscle parameters using recognized measurement methods (e.g., studies assessing only body mass index or general nutritional indicators).

(3)Studies that did not report mortality outcomes or did not provide sufficient data to estimate effect sizes.

(4)Reviews, systematic reviews, meta-analyses, conference abstracts, case reports, editorials, letters, or animal studies.

(5)Duplicate publications based on the same population; in such cases, the study with the largest sample size or the most complete data was included.

**Appendix 3.Quality assessment-Newcastle-Ottawa Quality Assessment Scale**

In our quality assessment scale, studies were scored across three categories: selection (up to 4 points), comparability (up to 2 points), and outcome of study participants (up to 3 points). Studies with a cumulative score ≥ 7 were considered to be of high quality, 4 to 6 as moderate quality, and 0 to 3 as low quality. Note: A study can be awarded a maximum of one star for each numbered item within the Selection and Outcome categories. A maximum of two stars can be given for Comparability

Selection

1) Representativeness of the exposed cohort

a) truly representative of the average ______________ (describe) in the community*

b) somewhat representative of the average _____________ in the community* c) selected group of users e.g. nurses, volunteers d) no description of the derivation of the cohort

2) Selection of the non-exposed cohort

a) drawn from the same community as the exposed cohort*

b) drawn from a different source

c) no description of the derivation of the non-exposed cohort

3) Ascertainment of exposure

a) secure record (e.g. surgical records)*

b) structured interview*

c) written self-report

d) no description

4) Demonstration that outcome of interest was not present at start of study a) yes*

b) no

Comparability

1)Comparability of cohorts on the basis of the design or analysis

a) study controls for _____________ (select the most important factor)* b) study controls for any additional factor* (This criterion could be modified to indicate specific control for a second important factor.)

Outcome

1) Assessment of outcome

a) independent blind assessment*

b) record linkage*

c) self report

d) no description

2) Was follow-up long enough for outcomes to occur

a) yes (select an adequate follow up period for outcome of interest)*

b) no

3) Adequacy of follow up of cohorts

a) complete follow up - all subjects accounted for*

b) subjects lost to follow up unlikely to introduce bias - small number lost - > ____ % (select an adequate %) follow up, or description provided of those lost)*

c) follow up rate < ____% (select an adequate %) and no description of those lost

d) no statement

# 2 Supplementary Figures and Tables

**Supplementary Table S1.** GRADE summary of findings for the association between sarcopenia assessment methods and mortality in patients with liver cirrhosis

| Outcome | study | Effect size (HR, 95% CI) | Risk of bias | Inconsistency | Indirectness | Imprecision | Publication bias | GRADE |
| --- | --- | --- | --- | --- | --- | --- | --- | --- |
| CT (SMI) | 5 | 1.88 (1.50–2.36) | Not serious | Not serious (I²=0%) | Not serious | Not serious | Undetected | High |
| CT (PMI/PMT/TPMT) | 4 | 3.19 (1.62–6.28) | Not serious | Serious (I²=52%) | Not serious | Serious (wide CI) | Undetected | Moderate |
| HGS | 3 | 2.21 (1.61–3.05) | Not serious | Not serious (I²=0%) | Not serious | Not serious | Undetected | Moderate |
| US | 3 | 0.94 (0.64–1.40) | Not serious | Serious (I²=80%) | Not serious | Serious (non-significant) | Possible | Low |
| DXA | 2 | 0.69 (0.50–0.95) | Not serious | Not serious (I²=18%) | Not serious | Serious (small sample size) | Possible | Low |

**Table S2** Quality assessment of observational studies using Newcastle–Ottawa Scale.

|  |  | **seiection** | | | | **Comparability** | **outcome** | | |  |
| --- | --- | --- | --- | --- | --- | --- | --- | --- | --- | --- |
| **Study** | **Year** | **Representative ness of the  exposed  cohort  (1 point)** | **Selection  of the non  exposed  cohort  (1 point)** | **Ascertainment  of exposure**  **(1 point)** | **Demonstration  that outcome of  interest was not  present at start  of study  (1 point)** | **Comparability  of cohorts on  the basis of the  design or  analysis  (2 point)** | **Assessment  of outcome**  **(1 point)** | **Was follow-up  long enough  for outcomes  to occur  (1 point)** | **Adequacy  of follow up  of cohorts  (1 point)** | **Quality**  **(9 point)** |
| Dae Hoe Gu | 2018 | 1 | 1 | 1 | 1 | 2 | 1 | 1 | 1 | 9 |
| Abhinav Anand | 2022 | 1 | 1 | 1 | 1 | 2 | 1 | 1 | 1 | 9 |
| Thyloor Kenchappa | 2023 | 1 | 1 | 1 | 1 | 2 | 1 | 1 | 0 | 8 |
| Sun C | 2025 | 1 | 1 | 1 | 1 | 2 | 1 | 1 | 0 | 8 |
| Rodge GA | 2023 | 1 | 1 | 1 | 1 | 1 | 1 | 1 | 0 | 7 |
| Moctezuma-Velazquez C. | 2019 | 1 | 1 | 1 | 1 | 2 | 1 | 1 | 1 | 9 |
| Cho Y.S. | 2021 | 1 | 1 | 1 | 1 | 2 | 1 | 1 | 0 | 8 |
| Hanai T | 2019 | 1 | 1 | 1 | 1 | 2 | 1 | 1 | 1 | 9 |
| Nishikawa et al. | 2021 | 1 | 1 | 1 | 1 | 2 | 1 | 1 | 1 | 9 |
| Ciocîrlan M | 2019 | 1 | 1 | 1 | 1 | 2 | 1 | 1 | 1 | 9 |
| Kikuchi N | 2021 | 1 | 1 | 1 | 1 | 2 | 1 | 1 | 1 | 9 |
| Eriksen CS | 2021 | 1 | 1 | 1 | 1 | 2 | 1 | 1 | 1 | 9 |
| Belarmino G | 2018 | 1 | 1 | 1 | 1 | 2 | 1 | 1 | 1 | 9 |
| Ayadi | 2023 | 1 | 1 | 1 | 1 | 1 | 1 | 1 | 0 | 7 |
| De Monte S | 2025 | 1 | 1 | 1 | 1 | 2 | 1 | 1 | 0 | 8 |

**Supplementary Table S3.** Characteristics of included studies and sarcopenia assessment methods.

| **author** | **year** | **country** | **cut off** | **Source of cut-off** | **Measurement site** | **Assessment domain** | **Calculation method** | **Unit** | **Exposure comparison and reference category** | **Exposure format** |
| --- | --- | --- | --- | --- | --- | --- | --- | --- | --- | --- |
| Dae Hoe Gu | 2018 | Korea | Men：PMTH<17.3 mm/m; women：PMTH<10.4 mm/m | Study-derived ROC analysis using SMI-sarcopenia | Right psoas muscle at the umbilical level | Muscle mass | Transverse psoas thickness/height | mm/m | SsPMTH-sarcopenia vs non-sarcopenia | dichotomous |
| Abhinav Anand | 2022 | India | Men：SMI<36.5 cm²/m², women：SMI<30.2 cm²/m² | Benjamin et al. (2017), Indian/Asian population-specific SMI cut-offs | CT measured at the mid-body of L3, including total abdominal skeletal muscle area | Muscle mass | Total L3 muscle area/height² | cm²/m² | SMI-defined sarcopenia vs no sarcopenia | dichotomous |
| Thyloor Kenchappa | 2023 | India | Men: PMI <356 mm²/m²；Women: PMI <295 mm²/m² | Study-specific normative cut-offs (<2 SD below sex-specific control mean) | Cross-sectional area of the right psoas muscle at the L3 vertebral level on axial CT | Muscle mass | Right L3 psoas area/height² | mm²/m² | PMI-defined sarcopenia vs non-sarcopenia | dichotomous |
| Sun C | 2025 | China | Men: SMI <46.96 cm²/m²；Women: SMI <32.46 cm²/m² | Tianjin criteria; X-tile-derived mortality cut-offs | Total skeletal muscle area at L3 on CT | Muscle mass | Total L3 muscle area/height² | cm²/m² | TJ-defined sarcopenia vs non-sarcopenia | dichotomous |
| Rodge GA | 2023 | India | Men: PMI <425.72 mm²/m²；Women: PMI <364.14 mm²/m² | Study-specific control cut-offs (mean−2 SD) | Total cross-sectional area of the right and left psoas muscles at L3 on axial CT | Muscle mass | Total bilateral L3 psoas area/height² | mm²/m² | PMI sarcopenia vs non-sarcopenia | dichotomous |
| Moctezuma-Velazquez C. | 2019 | Canada | Men: SMI <50 cm²/m²；Women: SMI <39 cm²/m² | Carey et al. (2017), multicenter mortality-based cut-offs | Total skeletal muscle area at L3 on transverse CT | Muscle mass | Total L3 muscle area/height² | cm²/m² | SMI-defined sarcopenia vs non-sarcopenia | dichotomous |
| Cho Y.S. | 2021 | South Korea | Men: SMI ≤50.0 cm²/m²；Women: SMI ≤39.0 cm²/m² | Carey et al. (2017); Bhanji et al. (2019) | Total skeletal muscle area at L3 on a portal-phase axial CT image | Muscle mass | Total L3 muscle area/height² | cm²/m² | SMI-defined sarcopenia vs no sarcopenia | dichotomous |
| Hanai T | 2019 | Japan | Men: HGS <30 kg；Women: HGS <15 kg | Study-specific ROC/Youden-derived mortality cut-offs | Bilateral handgrip strength measured in a standing position | Muscle strength | Mean of the highest HGS values from both hands | Kg | Low HGS vs high HGS | dichotomous |
| Nishikawa et al. | 2021 | Japan | Men: HGS <26 kg；Women: HGS <18 kg | JSH criteria based on AWGS 2014 | Bilateral handgrip strength measured in the standing position | Muscle strength | Mean of the highest HGS values from both hands | Kg | Muscle weakness vs normal HGS | dichotomous |
| Ciocîrlan M | 2019 | Romania | NR | NA | Bilateral rectus abdominis, approximately 2 cm above and 3 cm lateral to the umbilicus | Muscle thickness | Mean of bilateral maximum RA thickness | mm | Per 1-mm increase in mRA; no reference category | continuous |
| Kikuchi N | 2021 | Japan | Men：SMI≤42.0 cm²/m², women：SMI≤38.0 cm²/m²； | JSH 2016 criteria; muscle strength based on AWGS | total skeletal muscle area at L3 on CT | Muscle mass | Total L3 muscle area/height² | cm²/m² | SMI-defined sarcopenia vs no sarcopenia | dichotomous |
| Eriksen CS | 2021 | Denmark | NR | NA | Bilateral arm lean mass measured by whole-body DXA | Muscle mass | Arm lean mass/height² | kg/m² | Per 1-kg/m² increase in AI; no reference group | continuous |
| Belarmino G | 2018 | Brazil | NR | NA | Bilateral arms and legs lean mass by whole-body DXA | Muscle mass | Four-limb lean mass / height² | kg/m² | Per 1 kg/m² increase in DXA-ASMI | continuous |
| Ayadi | 2023 | Tunisia | TPMT/h <16.8 mm/m | Durand et al. (2014) | Psoas muscle at the umbilical level on axial abdominal CT； | Muscle thickness | Transverse psoas thickness/height | mm/m | TPMT/h sarcopenia vs non-sarcopenia | dichotomous |
| De Monte S | 2025 | German | MTRFM/height² <2.2 mm/m² | Study-specific ROC/Youden Index-derived cutoff for 6-month death or liver transplantation | Dominant-leg rectus femoris at one-quarter of the distance between the upper patella and anterior superior iliac spine | Muscle thickness | Mean RFM thickness of 3 measurements/height² | mm/m² | Low MTRFM/height² vs preserved MTRFM/height² | dichotomous |

**Supplementary Table S4.** Characteristics of adjusted and unadjusted effect estimates included in the meta-analysis

| **author** | **year** | **Assessment method** | **Cut off** | **HR type** | **Adjusted variables** | **effect estimate source** |
| --- | --- | --- | --- | --- | --- | --- |
| Dae Hoe Gu | 2018 | CT:PMTH | Men：PMTH<17.3 mm/m; women：PMTH<10.4 mm/m | Adjusted HR | Underlying liver disease, platelet count, INR, bilirubin, albumin, creatinine, sodium | Directly extracted |
| Abhinav Anand | 2022 | CT:SMI | Men：SMI<36.5 cm²/m², women：SMI<30.2 cm²/m² | Adjusted HR | Ascites | Directly extracted |
| Thyloor Kenchappa | 2023 | CT:PMI | Men: PMI <356 mm²/m²；Women: PMI <295 mm²/m² | Adjusted HR | NR | Multivariate analysis / Cox regression analysis |
| Sun C | 2025 | CT:SMI | Men: SMI <46.96 cm²/m²；Women: SMI <32.46 cm²/m² | Adjusted HR | Age, BMI (<25 kg/m²), MELD-Na score / CTP class | Multivariate Cox regression analysis |
| Rodge GA | 2023 | CT:PMI | Men: PMI <425.72 mm²/m²；Women: PMI <364.14 mm²/m² | Unadjusted HR | NR | Reported HR from survival analysis |
| Moctezuma-Velazquez C. | 2019 | CT:SMI | Men: SMI <50 cm²/m²；Women: SMI <39 cm²/m² | Adjusted HR | BMI; ascites; MELD; sodium; hepatic encephalopathy | Multivariable Cox proportional hazards model |
| Cho Y.S. | 2021 | CT:SMI | Men: SMI ≤50.0 cm²/m²；Women: SMI ≤39.0 cm²/m² | Adjusted HR | Age; sex; MELD score; HVPG | Directly reported from multivariable Cox proportional hazards regression model |
| Hanai T | 2019 | HGS | Men: HGS <30 kg；Women: HGS <15 kg | Adjusted HR | Age; cirrhosis etiology; HCC; serum sodium; BTR; Child–Pugh score; BMI; SMI; SATI; VATI | Directly reported from multivariable Cox regression model |
| Nishikawa et al. | 2021 | HGS | Men: HGS <26 kg；Women: HGS <18 kg | Adjusted HR | Female gender; CLD etiology; HCC presence; Child–Pugh class; muscle strength weakness | Directly reported from multivariate Cox proportional hazards model |
| Ciocîrlan M | 2019 | US | NR | Adjusted HR | Rectus abdominis thickness (mRA); ascites | Directly reported from multivariate Cox proportional hazards regression |
| Kikuchi N | 2021 | CT:SMI | Men：SMI≤42.0 cm²/m², women：SMI≤38.0 cm²/m²； | Adjusted HR | Age; sarcopenia; decompensated phase; HCC stage 3/4; ALBI grade; AST; eGFR; platelet count; hemoglobin; prothrombin time | Multivariate Cox proportional hazards regression |
| Eriksen CS | 2021 | DXA | NR | Adjusted HR | Weight; sodium; MAP; HVPG; Child-Pugh score | Multivariate Cox proportional hazards regression |
| Belarmino G | 2018 | DXA | NR | Adjusted HR | Age; MELD score | Multivariate Cox proportional hazards regression |
| Ayadi | 2023 | CT:L3-TPMT | TPMT/h <16.8 mm/m | Adjusted HR | Age; sex; variceal hemorrhage; hepatic encephalopathy; number of complications; hospitalization duration; | Multivariate Cox regression model |
| De Monte S | 2025 | US | MTRFM/height² <2.2 mm/m² | Adjusted HR | MELD score | Directly extracted from multivariate Cox regression |

Figure S1 Funnel plot and trim-and-fill analysis for assessment of publication bias.

Figure S2 Leave-one-out sensitivity analysis for the overall meta-analysis.
